# Therapeutic applications of eucalyptus essential oils

**DOI:** 10.1007/s10787-024-01588-8

**Published:** 2024-11-05

**Authors:** Riham A. El Shiekh, Ahmed M. Atwa, Ali M. Elgindy, Aya M. Mustafa, Mohamed Magdy Senna, Mahmoud Abdelrahman Alkabbani, Kawther Magdy Ibrahim

**Affiliations:** 1 Department of Pharmacognosy, Faculty of Pharmacy, Cairo University, Kasr El-Aini St., Cairo, 11562 Egypt; 2 Department of Pharmacology and Toxicology, Faculty of Pharmacy, Egyptian Russian University, Cairo, Egypt; 3 Department of Pharmacology and Toxicology, College of Pharmacy, Al-Ayen Iraqi University, Thi-Qar, 64001 Iraq

**Keywords:** Essential oils, *Eucalyptus*, Drug discovery, Biological activities, 1.8-Cineole

## Abstract

*Eucalyptus* essential oils (EEOs) have gained significant attention recently anticipated to their broad range of prospective benefits in various biological applications. They have been proven to have strong antibacterial properties against a variety of bacteria, fungi, and viruses. This makes them valuable in combating infections and supporting overall hygiene. The active compounds present in these oils can help alleviate inflammation, making them valuable in addressing inflammatory conditions such as arthritis, respiratory ailments, and skin disorders. Respiratory health benefits are another prominent aspect of EEOs. Inhalation of these oils can help promote clear airways, relieve congestion, and ease symptoms of respiratory conditions like coughs, colds, and sinusitis. They are often utilized in inhalation therapies and chest rubs. They can be used topically or in massage oils to alleviate muscle and joint pain. Furthermore, these oils have shown potential in supporting wound healing. Their antimicrobial activity helps prevent infection, while their anti-inflammatory and analgesic properties contribute to reducing inflammation and pain associated with wounds. In aromatherapy, EEOs are renowned for their invigorating and uplifting qualities, promoting mental clarity, relaxation, and stress relief. Overall, EEOs hold great promise in biological applications, offering a natural and versatile approach to promote health and well-being. Continued research and exploration of their therapeutic potential will further unveil their benefits and broaden their applications in various fields.

## Introduction

Essential oils (EOs) are extracted from various plant parts, such as wood, leaves, roots, flowers, and fruits, using steam or hydro-distillation techniques. These oils are complex combinations of terpenic chemicals, mainly monoterpenes and sesquiterpenes, along with alcohols, aldehydes, ethers, esters, ketones, and phenols, which contribute to their distinctive scents (Dhakad et al. 2018). The *Eucalyptus* species are comprising over 900 species and subspecies, is native to Australia and has been successfully cultivated in various regions with subtropical and Mediterranean climates (Cermelli et al. 2008; Okba et al. 2021). They are noted for their considerable biomass and strong essential oil content, which represents a significant source of natural chemicals with diverse and useful bioactive qualities (Ashour, et al. 2023; da Silva et al. 2020). *Eucalyptus* species are predominantly evergreen, fast-growing perennials that can reach impressive heights of 80 to 90 m. Their adaptability to diverse soils and climates has facilitated their widespread cultivation, with *Eucalyptus* plantations exceeding 22.57 million hectares globally as of 2022 (Zhang et al. 2010). They are utilized for a multitude of purposes, including the production of wood, fibers, cellulose, dyes, pulp, gum, resin, and EOs (Iglesias et al. 2021). In traditional medicine, they are valued for their efficacy in controlling pests like ticks and mosquitoes (Batish et al. 2008), as well as in treating respiratory ailments.

**Fig. 1:**
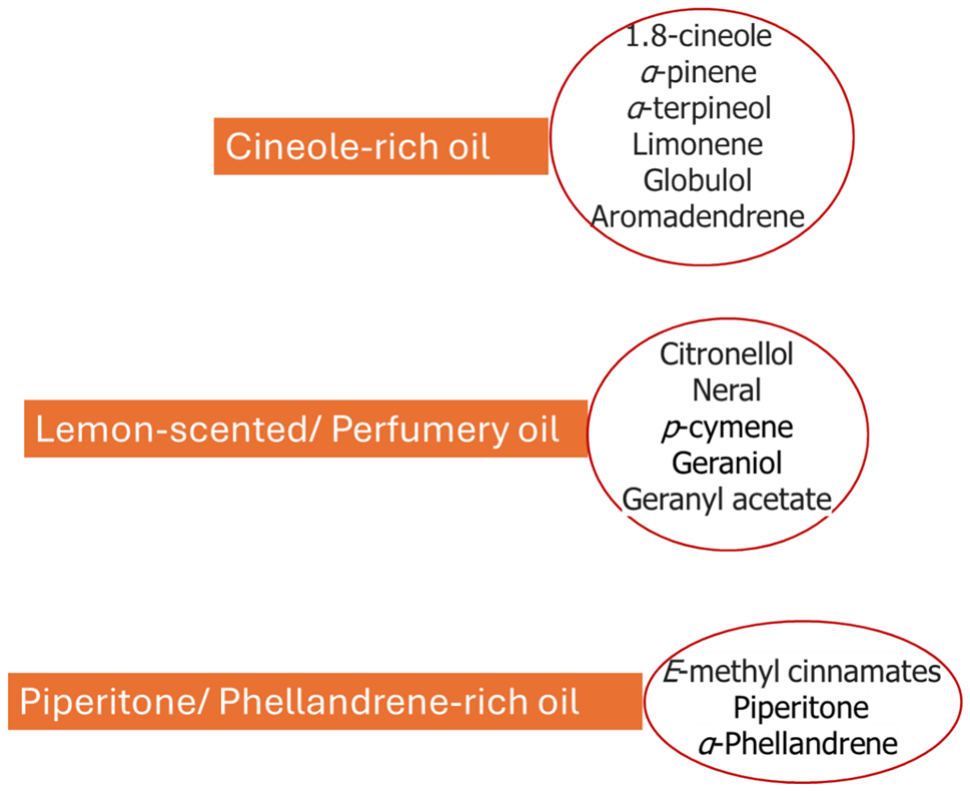
Different constituents of EEOs

Eucalyptus essential oils (EEOs) are primarily derived through the steam distillation or hydrodistillation of leaves, and less frequently from fruits, flowers, and stems (Bossou et al. 2015; Hamdi et al. 2015; Mubarak et al. 2015), with a chemical composition comprising a mixture of volatile bioactive compounds, primarily monoterpenoids, along with smaller quantities of sesquiterpenes (Mieres-Castro et al. 2021). Their high EO yields and diverse phytochemical profiles (Fig. 1) expand their utility beyond traditional public health and medicine applications, making them valuable in a variety of industries. In addition, EEOs are characterized by their ease of extraction, biodegradability, and low toxicity to vertebrates, placing them as environmentally beneficial options in many applications (Gilles et al. 2010). Notably, EOs from specific species, such as *E. polybractea*, *E. smithii*, and *E. globulus*, have garnered consideration for their applications in pharmaceuticals and cosmetics (Gilles et al. 2010; Manika et al. 2013; España et al. 2017).

Furthermore, the phytopharmacological potential of EEOs is well-documented, showcasing a broad spectrum of biological activities, including antibacterial (Ghalem and Mohamed 2008; Sabo and Knezevic 2019), antifungal (Nikbakht et al. 2015), antiviral (Mieres-Castro et al. 2021; El-Shiekh et al. 2024), anti-inflammatory (Salvatori et al. 2023), antioxidant (Harkat-Madouri et al. 2015), and wound healing (Alam et al. 2018) effects. These properties make EEOs particularly effective in addressing respiratory conditions, such as the common cold, sinusitis, bronchitis, and chronic obstructive pulmonary disease (Mieres-Castro et al. 2021).

Given their great toxicity in vapour form against a broad spectrum of microorganisms and insects, EEOs offer potential as commercially viable fumigants for laboratories, hospitals, food storage facilities, and other contexts (Dhakad et al. 2018). Although there is scant research on clinical trials that convincingly establish the therapeutic effects of EEOs and cineole, these chemicals are significant components of many proprietary treatments, including syrups, lozenges, nasal drops, and inhalation preparations (Gilles et al. 2010). Furthermore, essential oils derived from numerous Eucalyptus species are widely used in aromatherapy throughout Europe and Japan. Despite its obvious practical benefits and intrinsic therapeutic capabilities, there is an urgent need to evaluate the active components in EEOs for use in chemotherapy and other Ayurvedic treatments globally (Dhakad et al. 2018).

Numerous countries' pharmacopoeias, including the United States, Spain, the United Kingdom, Germany, France, Belgium, the Netherlands, Australia, Japan, and China, have acknowledged the medicinal benefits and applications of EEOs. These applications include infusion, inhalation (steam), and topical use. Among the Eucalyptus species, *E. globulus* is the most extensively employed in the phytopharmaceutical business for the manufacture of high-quality essential oils. This preference is primarily owing to the widespread cultivation of *E. globulus* globally and the genetic selection techniques used to optimize various wood production traits in breeding projects (Malakar 2024). The high 1,8-cineole concentration of *E. globulus* essential oil contributes to its powerful therapeutic capabilities, making it an important resource in the production of various pharmaceutical products and formulations. Pharmacopoeias use standardization and quality control procedures to ensure that Eucalyptus oil-based medicines are consistently effective and safe (Salehi et al. 2019a; Fitriani et al. 2020; Chandorkar et al. 2021).

EEOs have been used in traditional medicine for generations, and they are regarded for their ability to improve respiratory health, promote good skin, and relieve muscle tension. In recent years, drugs containing 1,8-cineole and standardized EEO extracts, such as Myrtol® capsules, have received attention for their therapeutic advantages in a variety of respiratory disorders. These products, marketed under names like GeloMyrtol® and GeloMyrtol forte®, typically contain a blend of 1,8-cineole, limonene, and α-pinene. While EEOs is commonly used in aromatherapy and topical applications, it is important to note that internal use should be done with caution and under the guidance of a healthcare professional. Proper dilution and skin testing are recommended before using Eucalyptus oil on the skin, as it may cause irritation in some individuals (Dontje et al. 2024).

By integrating this knowledge, the review offers a comprehensive overview of the diverse applications and benefits of EEOs, emphasizing their significance across various industries and their potential as sustainable, nature-based solutions to critical challenges in medicine, agriculture, and beyond.

Eucalyptus essential oil remains a significant component of traditional medicine, offering a range of therapeutic benefits that have been validated through both historical practices and modern research. Its applications extend beyond health to include personal care and industrial uses, highlighting its versatility and enduring relevance in various fields. As research continues to uncover the full potential of eucalyptus oil, its traditional uses will likely be further substantiated and expanded upon in contemporary practices. The use of Eucalyptus by Aboriginal Australians dates back thousands of years, where it was often prepared as infusions or poultices for medicinal purposes. The introduction of eucalyptus oil to Western medicine occurred in the late eighteenth century, with early distillation efforts aimed at treating respiratory issues among convicts in Australian settlements. Over time, the oil gained recognition not only for its medicinal properties but also for its versatility in industrial applications, including its use as a flavoring agent and in the production of cellulose pulp for paper manufacturing (Clarke 2008; Akhtar et al. 2016; Nangala et al. 2019).

## Search strategy

The databases utilized for this review include PubMed, ScienceDirect, SciFinder, Scopus, SciELO, and Google Scholar, all accessed on June 30, 2024. The primary search terms employed were “*Eucalyptus* essential oil + biological activities,” “*Eucalyptus* essential oil + chemical composition,” and “*Eucalyptus* oil + extraction methods.”

This review encompasses all articles that investigate the EOs derived from *Eucalyptus* species, as well as those detailing the composition of EEOs for medicinal applications.

Only experimental studies and reviews published in peer-reviewed publications before June 2024 were included. Articles written in languages other than English, theses, and unpublished data were also removed, as were research that were not directly relevant to Eucalyptus species. The data acquired from the selected articles were analyzed qualitatively, with a close evaluation of the content. After reading the full texts, papers that did not relate to EEOs or their core components were removed, as were those with restricted conclusions. Furthermore, the main articles were searched to locate additional relevant publications. This methodical approach aided in the selection of the most relevant publications that satisfied the eligibility requirements, summarizing EEO applications and biological activities for this literature study.

## Family Myrtaceae

The Myrtaceae family, commonly referred to as the myrtle family, represents a significant group of dicotyledonous woody plants within the order Myrtales. This family encompasses approximately 5,800 species distributed across 130 to 150 genera, making it the eighth largest flowering plant family globally. Members of the Myrtaceae are ecologically and economically important, thriving in diverse regions including South America, Australia, tropical Asia, Africa, and Europe (de Paulo Farias et al. 2020). Characterized by their rich array of bioactive phytochemicals, Myrtaceae species contain essential oils, terpenoids, carotenoids, phenolic acids, flavonoids, and phloroglucinols (Malakar 2024). These compounds have garnered considerable attention for their applications in food and pharmaceutical industries due to their potential health benefits. The chemical composition of Myrtaceae plants can vary significantly based on several factors including genus, species, geographical location, climate conditions, the specific plant part utilized, and the phenological stage at which they are harvested (Padovan et al. 2014). The medicinal and aromatic properties of Myrtaceae species are primarily attributed to their phenolic acids and flavonoids, which often exist in glycosylated forms within their fruits. This includes a variety of flavonoids such as flavanones, isoflavones, flavones, flavonols, flavanols, and anthocyanins. The phenolic acids are predominantly derived from hydroxycinnamic acid (C6–C3) and include compounds like *p*-coumaric acid, caffeic acid, hydroxybenzoic acid, vanillic acid, gallic acid, and ellagic acid (Celaj et al. 2021). Research has demonstrated that Myrtaceae species exhibit a diverse range of biological activities including antibacterial, anticoagulant, antifungal, antioxidant, cytotoxic, antiviral, anti-inflammatory, anti-parasitic, and antidiabetic effects (Macedo et al. 2021). These bioactivities are largely attributed to their major bioactive compounds such as phenolics and triterpenes found in essential oils (da Veiga Correia et al. 2022; Qamar et al. 2022; Santos et al. 2024). The mechanisms underlying these biological effects vary depending on the specific chemical constituents involved and the biological assays employed. Furthermore, extracts and essential oils derived from Myrtaceae plants have shown promise in various industrial applications including food production and cosmetics. The large-scale utilization of these extracts has led to innovative methods for extracting and purifying their bioactive compounds (Benvenutti et al. 2021).

## Eucalyptus species

Eucalyptus species, particularly *E. globulus* (Blue Gum), are renowned for their therapeutic applications. *E. globulus* is commonly utilized as an expectorant to alleviate symptoms associated with respiratory conditions such as bronchitis and asthma. Its efficacy extends to treating mild inflammation of the respiratory tract, making it a staple ingredient in cough syrups and lozenges. The major component, 1,8-cineole (eucalyptol), is recognized for its anti-inflammatory properties and is often incorporated into mouthwashes and topical formulations for pain relief (Collins et al. 1990a, 1990b). Additionally, this species exhibits antibacterial properties, enhancing its utility in treating infections. Another notable species, *E. citriodora* (Lemon Eucalyptus), is primarily valued for its insect-repelling qualities due to high levels of citronellal. This species finds applications in perfumery and cosmetics and is also used in aromatherapy for relaxation and stress relief (Collins et al. 1990a, 1990b). Similarly, *E. radiata* serves a comparable role to *E. globulus*, being effective for respiratory ailments while providing a milder aroma that makes it suitable for aromatherapy. *E. staigeriana* (Lemon-scented Gum) is celebrated for its uplifting scent and is frequently employed in aromatherapy to alleviate stress and anxiety. It contains compounds that may offer antimicrobial benefits (Collins et al. 1990a, 1990b). Meanwhile, *E. polybractea* (Blue Mallee), rich in 1,8-cineole, is similarly used for respiratory issues and its essential oil is applied topically to relieve muscle pain. Other species such as *E. dives* (Broad-leaved Peppermint) are recognized for their astringent properties and are traditionally used to treat fevers by inhaling smoke from burned leaves (Collins et al. 1990a, b). *E. viminalis* (Manna Gum) is utilized for treating ophthalmia and gastrointestinal issues like diarrhea, with its leaves containing eucalyptol known for its antiseptic effects. *E. camaldulensis* (River Red Gum) is valued for its disinfecting properties, traditionally used in remedies for cuts and sores, while the sap has been historically employed to treat diarrhea in children. Also, *E. saligna* (Sydney Blue Gum) has been traditionally used for its anti-inflammatory properties and serves as a general tonic, contributing to the diverse therapeutic landscape of eucalyptus species (Collins et al. 1990a, 1990b). Collectively, these species highlight the significant role of eucalyptus in traditional and modern medicine, underscoring their varied applications across respiratory health, antimicrobial treatments, and holistic wellness practices.

## Yield of EEOs

Various analytical techniques, including HPLC, GC–MS, GC–EIMS, GC–FID, UV–Vis, and H-NMR, have been utilized to characterize the chemical profile of EEOs. The reported oil yield for *Eucalyptus* species in the literature exhibits considerable variability, ranging from 0.07 to 9% on a dry weight basis. The yield of EEOs extracted varies significantly depending on the species and organs. Several factors influence these yields, including seasonal fluctuations, tree age, leaf age, altitude, season, harvest timing, and fertilizer application (Jinbiao et al. 2010; Danna et al. 2023).

## Biodiversity of EEO components

Essential oils are a collection of volatile chemicals that give plants a particular scent. They are chemically composed of a complex blend of terpenoids, including monoterpenes (C10) and sesquiterpenes (C15), as well as other groups of molecules such as phenols, oxides, ethers, alcohols, esters, aldehydes, and ketones (Rodilla et al. 2024). There are about 900 species in the genus *Eucalyptus* L'Héritier, and more than 300 of those have volatile essential oils in their leaves. Of them, about 20 species have a 1,8-cineole concentration over 70%, which makes them economically viable for the manufacture of essential oils for the cosmetic and pharmaceutical industries (Silva et al. 2011). Certain plant anatomical features, including trichomes, idioblasts, oil ducts, and oil glands, are responsible for the production and storage of essential oils (EOs). Oil glands in the foliar mesophyll of the Eucalyptus species are the main source of EOs, however they are also found in the stems, flowers, and seeds. Therefore, the aerial parts of the plant are usually removed using steam distillation or hydro-distillation to extract EEOs (Danna et al. 2024). Numerous internal and external factors, such as species diversity, plant age, the physical and chemical characteristics of the growth environment, and harvesting circumstances like season, location, climate, soil type, and developmental stage, all have an impact on the chemical composition of these essential oils. The techniques used to extract and characterize the EOs also have an impact on their chemical compositions. These factors work together to influence the final spectrum of chemicals found in the essential oils (Afshari and Rahimmalek 2021). EEOs can be divided into different groups according to their chemotypes. The main ingredient in the majority of EEOs is 1.8-cineole, sometimes referred to as eucalyptol. Monoterpenes and sesquiterpenes such as α-pinene, p-cymene, α-terpineol, limonene, γ-terpinene, α-phellandrene, β-pinene, globulol, aromadendrene, and β-phellandrene are also noteworthy components. Furthermore, additional chemicals have been found, including viridiflorol, spathulenol, α-eudesmol, o-cymene, 4-terpineol, piperitone, and pinocarveol. Interestingly, although 1.8-cineole is the most prevalent main component in many species, in some Eucalyptus species, such *E. staigeriana* and *E. elata*, it is not the predominant constituent. Furthermore, in species such as *E.* globulus, different populations within the same species may have different concentrations of different chemicals due to a variety of causes (Barbosa et al. 2016). *E. globulus* EOs, commonly referred to as blue gum, is a well-liked and adaptable oil with a broad variety of medicinal uses. The main method of extraction is steam distillation of the Australian-native eucalyptus tree's leaves and twigs. The primary active ingredient in the oil is 1,8-cineole, commonly known as eucalyptol, which gives it its distinctively energizing and revitalizing scent (Smith 2023).

**Fig. 2:**
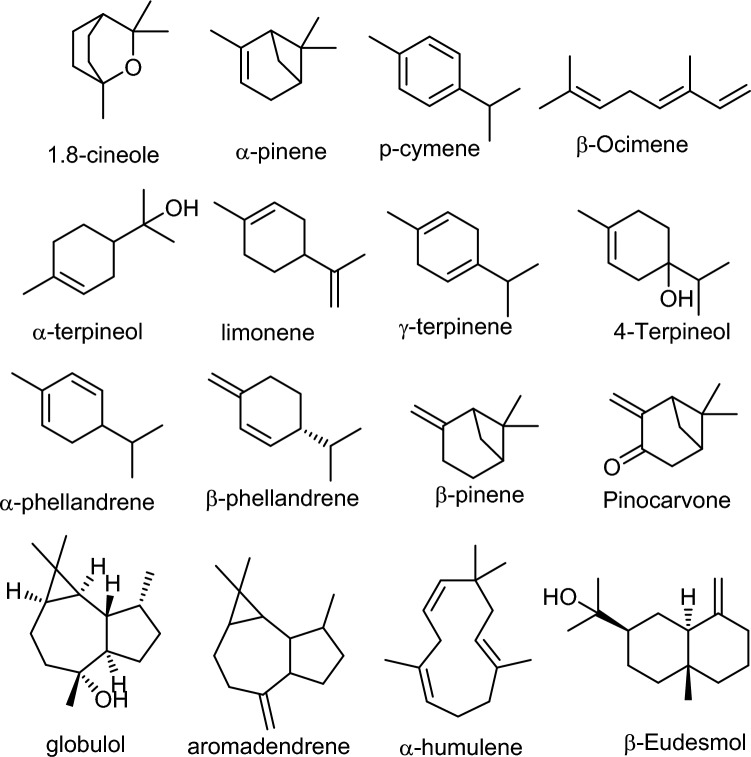
The major constituents of the EEOs in the analyzed literature

The chemical biodiversity of eucalyptus essential oils is a testament to the remarkable adaptability and diversity of the *Eucalyptus* genus. This chemical richness offers opportunities for the development of novel therapeutic agents, natural insecticides, and other value-added products. Different *Eucalyptus* species have diverse compositions of bioactive chemicals, which results in differences in their therapeutic qualities. The main species of *Eucalyptus* used medicinally are listed in Table 1, together with the percentages of each key ingredient and the chemical makeup of their essential oils. However, further research is needed to fully harness the potential of this chemical diversity and to optimize the applications of eucalyptus essential oils in various industries. The chemical structures of the important monoterpenes and sesquiterpenes are also shown in Fig. 2.

**Table 1:** The top ten *Eucalyptus* species used for medicinal uses with chemical composition, and the total percentage of compounds of their essential oils

Species	Constituents%	Refs
*E. globulus*	1.8-Cineole (83.0), limonene (9.0), α-pinene (4.0), *p*-cymene (3.0)	Santos et al. (2021)
*E. leucoxylon*	1,8-Cineole (77.76), α-pinene (5.85), trans-pinocarveol (3.23), globulol (1.42), limonene (1.33), pinocarvone (1.15)	Sebei et al. (2015)
*E. polybractea*	1.8-Cineole (85.01), *p*-cymene (4.12)	Juan et al. (2011)
*E. dives*	piperitone (47.87), α-phellandrene (23.33)	Demeter et al. (2021)
*E. Radiata*	1.8-Cineole (68.36), α-terpineol (12.44), limonene (7.32)	Juan et al. (2011)
*E. Smithii*	1,8 Cineole (78.49), limonene (5.88), β-eudesmol (5.46)	Juan et al. (2011)
*E. maidenii*	1.8-Cineole (66.71), α-pinene (10.34), α-terpineol (7.38)	Sadraoui-Ajmi et al. (2022)
*E. bicostata*	1.8-Cineole (63.00), α-pinene (14.4), limonene (10.9)	Roh et al. (2013)
*E. camaldulensis*	Spathulenol (38.9), *p*-cymene (19.05)	El-Shiekh et al. (2024)
*E. sideroxylon*	1.8-Cineole (55.9), α-phellandrene (10.6)	El-Shiekh et al. (2024)

## Biosynthetic pathways

EOs comprise two distinct groups of compounds, each with unique biosynthetic origins, that are primarily responsible for their biological properties. The major constituents can be categorized into terpenes and terpenoids. Terpenes are formed from combinations of several 5-carbon units known as isoprene, while terpenoids are oxygenated derivatives of terpenes. The predominant hydrocarbon terpenes in EOs can constitute over 80% of the oil and include monoterpenes (C10) and sesquiterpenes (C15). These compounds exhibit a variety of structural forms, including acyclic, mono-, bi-, and tricyclic structures. Terpenoids present in EOs encompass a wide range of chemical functionalities, including alcohols, aldehydes, ketones, acids, phenols, ethers, and esters (Baptista-Silva et al. 2020). While terpenes and phenylpropanoids generally follow different biosynthetic pathways and are often separated within plant tissues, some species may contain both groups, with one pathway predominating. In aromatic plant species, the biosynthesis of essential oils occurs through two intricate biochemical pathways that involve various enzymatic reactions. The universal precursors for essential oil biosynthesis are isopentenyl diphosphate (IPP) and its isomer dimethylallyl diphosphate (DMAPP). These compounds are generated via the cytosolic mevalonic acid (MVA) pathway or through the plastidic 1-deoxy-D-xylulose-5-phosphate (DXP) pathway, also known as the 2-C-methylerythritol 4-phosphate (MEP) pathway. Within specific plant cell compartments, prenyl diphosphate synthases further condense IPP and DMAPP to produce prenyl diphosphates, which serve as substrates for geranyl diphosphate (GPP, C10) or farnesyl diphosphate (FPP, C15). Essential oils, which are the final terpenoid products, are synthesized by a diverse group of enzymes known as terpene synthases (TPS). Essential oils are significant secondary metabolites in plants and have been utilized in various industries as well as in traditional ethnobotanical medicine for centuries. Consequently, extensive research has been conducted to elucidate the biosynthetic pathways of essential oils. This review aims to provide valuable insights into the biosynthesis of essential oils in plants, highlighting new and unexplored avenues for future research in the field of natural products (Rehman et al. 2016). The formation of geraniol, an acyclic monoterpene, is primarily facilitated through the chemical modification of two key precursors: geranyl pyrophosphate (GPP) and neryl pyrophosphate (NPP). Understanding the biochemical pathways leading to the synthesis of geraniol involves exploring the roles of phosphatases, particularly GDPases, in hydrolyzing these precursors into their corresponding alcohols. Acyclic monoterpenes like geraniol are derived from GPP or NPP through specific enzymatic modifications. In contrast, cyclic monoterpenes such as limonene, α-pinene, and β-pinene are generally produced from NPP. GPP cannot directly cyclize due to steric hindrance, making it less likely to form cyclic structures. Phosphatases play a crucial role in the hydrolysis of GPP and NPP. This process involves the removal of pyrophosphate groups, resulting in the formation of geraniol from GPP and other alcohols from NPP. Previous studies have documented this phosphatase-mediated hydrolysis across various plant species, underscoring its significance in terpenoid biosynthesis (Banthorpe et al. 1972; Croteau and Karp 1979; Pérez et al. 1980).

**Fig. 3:**
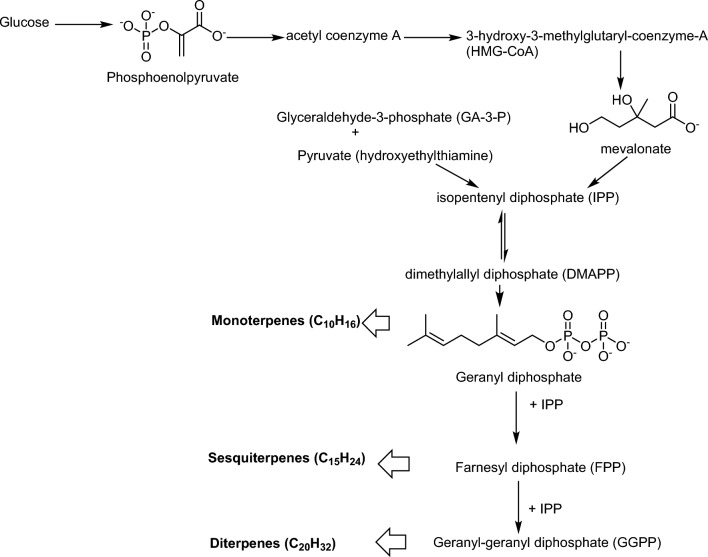
Terpenoid biosynthesis in the plants

The biosynthesis pathway of the terpenes in the aromatic plants is schematized in Fig. 3.

## Physicochemical properties of EEOs

EEOs present a captivating pale-yellow hue, exuding a pleasant and invigorating aroma reminiscent of camphor. This distinctive scent not only defines its olfactory profile but also contributes to its wide range of applications in various industries, including flavoring, fragrance, and cosmetics (Boukhatem et al. 2014; Harkat-Madouri et al. 2015; Benabdesslem et al. 2020). The predominant constituent of eucalyptus essential oil is 1,8-cineole, commonly referred to as Eucalyptol. This monoterpene oxide is celebrated for its aromatic qualities and is extensively utilized in flavoring agents, fragrances, and cosmetic products due to its pleasant spicy scent and taste. Furthermore, Eucalyptol finds applications in oral hygiene products such as mouthwashes and cough suppressants, underscoring its therapeutic potential (Damjanović-Vratnica et al. 2011; Mulyaningsih et al. 2011). The physical properties of this essential oil are critical for its quality assessment. The refractive index ranges from 1.4657 ± 0.0070 to 1.4693 ± 0.00057, indicating its purity and composition. The specific gravity falls between 0.913 and 0.919 g/cm^3^, which is essential for determining the oil's density and potential uses in formulations. Additionally, the optical rotation measures + 1.5956, reflecting the oil's chiral nature, while the pH at 22 °C is recorded at 4.9, indicating a slightly acidic character (Boukhatem et al. 2014). The chemical properties of the eucalyptus essential oil further elucidate its quality and functionality. The acid value is measured at 0.5945, indicating low acidity, while the saponification value stands at 19.576, which is important for understanding the oil's ability to form soaps and emulsions. The hydroxyl value is recorded at 31.61, suggesting the presence of alcohol that can influence solubility and reactivity. Lastly, the iodine value of 41.52 provides insight into the degree of unsaturation within the oil, which can affect stability and shelf life (Boukhatem et al. 2014). Collectively, these physicochemical properties are paramount for determining oil quality and are integral to effective marketing strategies. In terms of pharmacokinetics, EEOs are absorbed through various routes including inhalation, dermal application, and oral ingestion. Its bioactive compounds undergo metabolism primarily in the liver, with elimination occurring via urine and feces (Chandorkar et al. 2021). Understanding these physicochemical and pharmacokinetic attributes is vital for optimizing the therapeutic efficacy of eucalyptus essential oils in clinical settings.

## Biological activities of EEOs

**Fig. 4:**
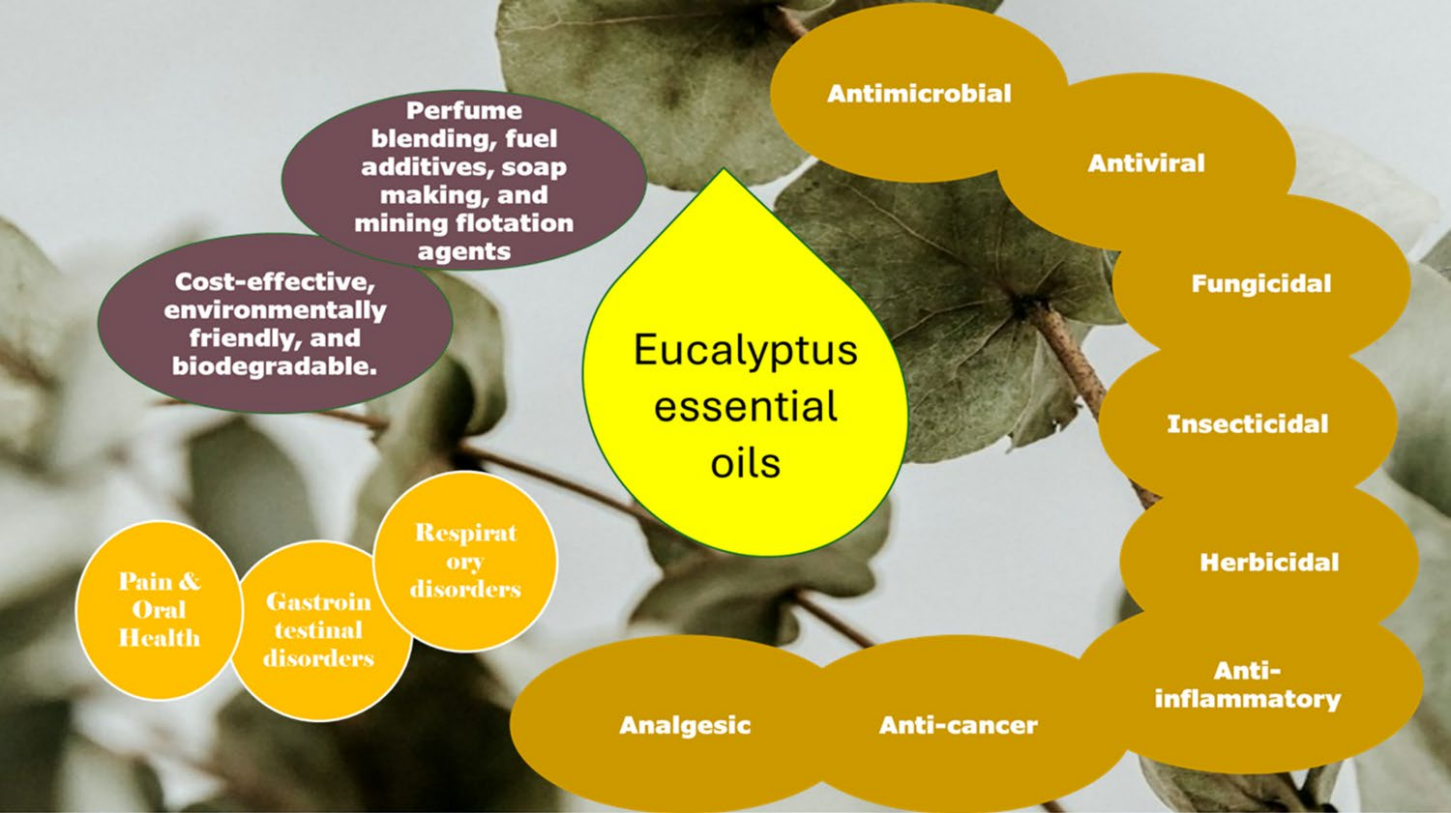
Biological activities of Eucalyptus essential oils (EEOs)

EEOs have attracted considerable attention due to their wide diversity of potential advantages in several biological applications and therapeutic characteristics (Fayez et al. 2023). These oils include chemical compounds with antibacterial (Elangovan and Mudgil 2023), anti-inflammatory (Salvatori et al. 2023), respiratory (Abbass 2020), pain-relief (Agarwal and Bhargava 2020), wound-healing (Mohammed et al. 2022), and aromatherapy properties (Robinson 2020), emphasizing their significance in supporting health and well-being **(**Fig. 4**)**.

Essential oils derived from *Eucalyptus* possess powerful antimicrobial characteristics, rendering them efficacious against an extensive array of bacteria, fungi, and viruses (Ahmad et al. 2023). This action is mostly due to the high cineole concentration (Tine et al. 2020; Polito et al. 2023). EEOs has been proven to suppress the development of a variety of pathogenic microbes, including *Staphylococcus aureus* (Qasim et al. 2021; Elangovan and Mudgil 2023; Fayez et al. 2023; Iseppi et al. 2023), *Escherichia coli* (KAREEM et al. 2020; Song et al. 2022), and *Candida albicans* (da Silva Gündel et al. 2020; Wunnoo et al. 2021; Fayez et al. 2023).

EEOs' anti-inflammatory characteristics make them useful for treating inflammatory conditions, including arthritis (Varkaneh et al. 2022; Ashour et al. 2023; Iqbal et al. 2024), respiratory problems (Smruti 2021; Her et al. 2022), and skin conditions (Almeida et al. 2022; Moreira et al. 2022; Takagi 2024). Moreover, EEOs contains active components such as cineole and alpha-pinene, which contribute to decrease inflammation by suppressing the generation of pro-inflammatory cytokines (Zhao et al. 2023).

The beneficial properties of EEOs on respiratory health are among their most notable effects (Li et al. 2023). Inhaling EEOs can facilitate the clearing of airways, alleviate obstruction, and alleviate the symptoms of respiratory conditions, including sinusitis, colds, and coughing (Sadlon and Lamson 2010; Soleimani et al. 2021; Her et al. 2022). The decongestant properties of cineole facilitate the breakdown of mucus and sputum, thereby facilitate their expulsion (Akkara et al. 2023). Consequently, EEOs are frequently utilized in cough preparations, chest rubs, and inhalation therapies (Abbass 2020; Stinson et al. 2023). Furthermore, the potential of EEOs to mitigate symptoms associated with COVID-19 has been investigated, predominantly due to its antiviral, anti-inflammatory, and decongestant properties. Symptomatic alleviation for individuals afflicted by the virus may be achieved through the inhalation of EEOs, which may alleviate respiratory symptoms such as congestion and wheezing (Abbass 2020; Sharma 2020; Panikar et al. 2021; KHAIRIAH et al. 2022; Lee et al. 2023).

EEOs are also effective in alleviating muscle and joint discomfort. They can mitigate the discomfort associated with conditions such as rheumatism, sprains, and muscle strains when administered topically or incorporated into massage oils (Yin et al. 2020; Varkaneh et al. 2022; Bakó et al. 2023; LO et al. 2024). EEOs 's analgesic properties may be attributed to its ability to desensitize nerve terminals and decrease the perception of pain (Silva et al. 2003). This makes it a favored option among athletes and individuals with chronic pain conditions (Lee et al. 2019; Chandorkar et al. 2021; Ridouh and Hackshaw 2022).

The wound-healing potential of EEOs is another significant application. Their analgesic and anti-inflammatory properties reduce inflammation and pain, while their antimicrobial activity aids in the prevention of infections in incisions (De Luca et al. 2021; Mohammed et al. 2022; Saeedi et al. 2023).

### Liver health

EEOs, particularly their active component eucalyptol, have demonstrated substantial potential in promoting liver health through various mechanisms, such as their antioxidant and anti-inflammatory capacities. Due to these properties, EEOs effectively mitigate hepatotoxicity caused by acetaminophen by significantly decreasing serum levels of hepatic enzymes and oxidative stress markers while increasing antioxidant enzyme activities and reducing inflammatory cytokines (Esmail et al. 2024).

In a lipopolysaccharide-induced inflammation model, EEOs suppressed inflammation by increasing antioxidant enzyme activities such as SOD and decreasing levels of TNF-α and NF-κB. This supports EEOs 's efficacy in treating hepatic inflammation and oxidative stress (Zhao et al. 2021). Additionally, EOs of *E. camaldulensis* reduced liver damage caused by carbon tetrachloride (CCl_4_) in Wistar rats. The oils exhibited hepatoprotective potential by preventing elevated serum ALT and AST levels, total cholesterol, triglycerides, and LDL-cholesterol, while significantly increasing plasma HDL-cholesterol levels and antioxidant enzyme activities, underscoring their antioxidant properties (Noumi et al. 2022). The hepatoprotective and nephroprotective effects of α-pinene, a compound found in *Eucalyptus*, have also been observed in rats exposed to CCl_4_. By reducing intracellular reactive oxygen species (ROS), α-pinene restored its antioxidant status, highlighting its potential for liver and kidney protection (Udavant et al. 2023).

Eucalyptol has demonstrated protective properties against cisplatin-induced liver injury in rats through the reduction of oxidative stress and DNA damage. Additionally, it enhances the function of antioxidant enzymes, including catalase and glutathione, and lowering MDA levels. Its hepatoprotective effects were further enhanced by the downregulation of iNOS expression (Akcakavak et al. 2023). *E. camaldulensis* extract protected the livers of mice against *Plasmodium chabaudi* infection. The extract improved liver histology and enzymatic activity alterations, prevented oxidative damage, and modulated the production of liver cytokines and interleukins, indicating its antioxidant and anti-inflammatory properties (Aljawdah et al. 2022).

In HepG2 cells, α-pinene increased cell viability, decreased oxidative stress indicators, and regulated inflammatory pathways, indicating its potential for controlling hyperglycemia-induced liver damage (Choghakhori et al. 2024). Furthermore, eucalyptol has been shown to lower oxidative stress and ROS generation, thereby downregulating the NF-κB and RIPK1 pathways and reducing cell necroptosis and inflammation in grass carp liver cells caused by chlorpyrifos (Yang et al. 2024). In the same cells, eucalyptol also protected hepatocytes from avermectin-induced injury by alleviating oxidative stress and reducing the expression of NLRP3 inflammasome-related genes (Wang et al. 2023).

### Cardiovascular system health

EEOs exhibit significant cardioprotective properties through various active constituents, each contributing to cardiovascular health via unique mechanisms. The prominent component, 1,8-cineole (eucalyptol), has been demonstrated to alleviate cardiovascular diseases, including hypertension and type 2 diabetes. Research conducted on rats and H9c2 cardiomyocytes has demonstrated that 1,8-cineole reduces ER stress-related apoptosis by modifying the miR-206-3p/SERP1 trajectory, thereby attenuating cardiac hypertrophy in heart failure. This underscores its potential to manage cardiovascular conditions by targeting specific molecular pathways (Oboh et al. 2013; Wang et al. 2021). Additionally, It ameliorates hypertrophy and enhances cardiac function in models of right ventricular hypertrophy and pulmonary arterial hypertension by reducing mitophagy, restoring mitochondrial respiratory capacity and homeostasis, and enhancing mitochondrial dynamics, all without causing toxicity (Alves-Silva et al. 2022). Furthermore, it shows potential for treating immune-thrombosis in conditions like COVID-19 and sepsis by inhibiting platelet activation and aggregation through activation of the AC-cAMP-PKA pathway. Moreover, it selectively inhibits glycoprotein VI-induced platelet activation and thrombus formation, reduces inflammatory cytokines, and may be useful for controlling excessive platelet reactivity (Alatawi 2021; Petry et al. 2024).

Eucalyptol has been shown to have a variety of cardiovascular benefits. In chronic nicotine-induced hypertension, it decreases plasma levels of MMP-9 and TIMP-1 markedly, suggesting its potential to mitigate cardiovascular complications (Murad 2023). Eucalyptol also enhances renal function and prevents the formation of atheromatous lesions in atherosclerotic models by reducing glycation, oxidative stress, and inflammatory mediators (Mahdavifard and Nakhjavani 2021). Furthermore, eucalyptol has been discovered to decrease myocardial contractility in a concentration-dependent manner. This property can be reversed by Ca^2+^ and isoproterenol, indicating that it plays a role in the management of myocardial contractility and the reduction of cardiac stress (Soares et al. 2005). Besides, its combined use with nifedipine has demonstrated enhanced effects in vasopressin-induced ischemia models, increasing coronary flow and angina symptoms while lowering oxidative and inflammatory markers (Murad and Alqurashi 2024).

Limonene, an additional significant constituent, provides numerous cardiovascular advantages. ( +)-Limonene is essential for the treatment of conditions such as hypertension and cardiac arrhythmia, as it promotes smooth muscle relaxation in the superior mesenteric artery of rats (de Sousa et al. 2015). The cardioprotective effects of D-limonene have been demonstrated in infarcted hearts of mice by reducing oxidative stress, restoring SOD and GPx activity, and suppressing pro-apoptotic pathways. Additionally, s-limonene mitigates cardiac remodeling in myocardial infarction models by reducing oxidative stress and Ca^2+^ overload, an observation that is comparable to the effects of well-known antioxidants such as N-acetyl cysteine (Durco et al. 2019; Rhana et al. 2022). D-Limonene has been demonstrated to significantly reduce myocardial infarction injury, primarily through the inhibition of the MAPK/NF-κB pathway, which affects subsequent transcriptional regulation. Furthermore, D-limonene's anti-apoptotic properties enhance its overall cardioprotective pharmacological effects (Younis 2020). Also, D-limonene mitigates cardiac toxicity caused by CCl_4_ intoxication by enhancing the antioxidant machinery, attenuating lipid peroxidation, and inhibiting inflammatory cascades through its antioxidant and anti-inflammatory properties (AlSaffar et al. 2022). In addition, l-limonene has the potential to enhance the quality of life for diabetic patients by modulating the AMPK/SIRT1/p65 signaling pathway, which also demonstrates anti-atherosclerotic properties in diabetic rats (Han et al. 2023).

Alpha-pinene, contained in EEOs, is also beneficial to cardiovascular health. It has cardioprotective and anti-inflammatory effects in isoproterenol-induced myocardial infarction models by decreasing cardiac marker enzyme levels, lipid peroxidation, and inflammatory mediators while restoring antioxidant status (Zhang et al. 2020). Furthermore, alpha-pinene and its metabolites, myrtenol and verbenol, greatly relax the blood vessels, mediated via endothelial-derived NO and guanylyl cyclase activity, emphasizing its vascular advantages (Jin et al. 2023). Moreover, monoterpenes α- and β-pinene inhibit bacterial growth in endocarditis, though they face resistance from strains such as *Staphylococcus aureus* (Salehi et al. 2019b).

Furthermore, *E. torquata* seeds extract (ETS) has demonstrated cardio-preventive properties against myocardial infarction in rats by significantly lowering cardiotoxicity, oxidative stress, and DNA fragmentation while improving lipid levels (Tej et al. 2024). *E. globulus* leaf extract has been shown to improve cardiac function in diabetic rats by rectifying oxidative stress, dyslipidemia, and cardiomyocyte depletion, as well as enhancing the expression of key cardiac proteins (Akinmoladun et al. 2023).

### Central nervous system

EEOs contains main components such as 1,8-cineole, α-pinene, and limonene. These compounds have antioxidant, anti-inflammatory, and neuroprotective properties. The central nervous system activities of the oil are primarily attributed to 1,8-cineole (Eucalyptol) and α-pinene (Sebei et al. 2015). Studies suggest that EEOs could be a promising therapy for neurodegenerative diseases such as Alzheimer's and Parkinson's by reducing neuroinflammation, modulating neurotransmitter systems, and promoting neuronal survival. Additionally, EEOs has shown anxiety-reducing and cognitive-enhancing effects (Qneibi et al. 2024). Furthermore, it can easily crosses the blood–brain barrier (BBB), regulating brain receptors and enzymes (Xu et al. 2021).

### Memory retention, neuroprotection, and Alzheimer’s disease

Memory loss disrupting daily life may indicate early dementia or Alzheimer's disease, marked by difficulty performing tasks and remembering dates. Alzheimer's is linked to amyloid plaques, neurofibrillary tangles, and neural connection loss in the brain (Soares et al. 2021). Alzheimer’s is also associated with decreased acetylcholine levels due to high activity of acetylcholinesterase and cholinergic neuron deterioration, leading to memory loss, a prominent symptom of the disease. *E. globulus* possesses anti-inflammatory and antimicrobial properties, in addition, it inhibits acetylcholinesterase (AChE), the enzyme that degrades the neurotransmitter acetylcholine into choline and acetate. This inhibition increases acetylcholine levels and prolongs its effects in the central nervous system, enhancing memory as well as preventing and slowing the progression of neurodegenerative diseases like Alzheimer. (Ayaz et al. 2017; Soares et al. 2021; Adedamola et al. 2023).

### Kidney health

Experimental models have demonstrated the efficacy of *E. globulus* in protecting several organs against redox imbalance (Dhibi et al. 2014). Impaired antioxidant system and oxidative stress have been recognized as crucial mechanisms in the pathogenesis of several illnesses. The presence of elevated amounts of free radicals in the system results in the decline of renal function and subsequently gives rise to tubulointerstitial fibrosis (Ganesan et al. 2018). The acute overdose of acetaminophen disrupted the equilibrium between oxidants and antioxidants, therefore disturbing redox homeostasis and inducing oxidative damage in the kidney. Administration of eucalyptus successfully prevented nephrotoxicity caused by acetaminophen by restoring nearly normal levels of superoxide dismutase (SOD), catalase (CAT), and glutathione (GPX) activity (Dhibi et al. 2014).

### Effects on skin and hair

EEOs with antibacterial qualities obtained from *Eucalyptus*, contain a significant number of terpenes, especially limonene, that exhibit great potential as bioactive components for optimizing hair and skin health. Studies indicated that the phytochemical components of EEOs, when applied topically, can interfere with the functional capacity of skin cells. Furthermore, these phytochemicals exert advantageous effects on anti-aging, anti-acne, sunscreen, and skin-lightening (Aswandi et al. 2023). EEOs exhibits anti-inflammatory, antioxidant, antibacterial, and antifungal properties, which strengthen the capillary fibers in the hair as well as promote health and cleanliness of the scalp (Zhou et al. 2021).

The antioxidant characteristics of EEOs can mitigate oxidative stress, which is a contributing factor to alopecia. Furthermore, EEOs significantly improve blood flow in the hair follicles as well as preserve the vascularization of hair epidermis papillae (Guzmán and Lucia 2021; Abelan et al. 2022; Aswandi et al. 2023). Incorporating EEOs into anti-dandruff haircare products boosts their effectiveness by combining anti-inflammatory, antifungal, and antioxidant properties, helping to eliminate the root causes of dandruff. Additionally, the use of EEOs benefits hair care by addressing seborrhea, a condition resulting from capillary dysfunction and oily hair fibers. This condition is partially alleviated by essential oils (Abelan et al. 2022).

### Dermato-protection effects

Effective cosmetic products containing *E. globulus* essential oils are crucial for improving skin hydration and maintaining overall skin health by supporting the skin barrier function, which can be disrupted by external and internal factors. Frequent use of soaps, detergents, hot water, or alcohol can remove surface lipids, leading to skin barrier damage and increased trans-epidermal water loss (TEWL) (Infante et al. 2022). The dermato-protective properties of *E. globulus* were investigated, showing notable inhibitory effects on tyrosinase. Moreira et al. (2022) investigation examined the potential protective properties of the essential oil and hydro-distillation residual water extract derived from E. globulus leaves against skin injury. The results revealed a decrease in age-related indicators of aging, such as the increased expression of type I collagen and the stimulation of matrix metalloproteinases and β-galactosidase. Likewise, there was reported suppression of melanin and tyrosinase synthesis (Assaggaf et al. 2022).

Elevated concentrations of pro-inflammatory cytokines and reactive oxygen species have a substantial role in the process of skin aging. A recent study revealed that an ethanolic extract derived from dehydrated commercial *E. globulus* biomass has a capacity to provide protection against UV-induced photoaging, therefore significantly diminishing the development of wrinkles and mitigating skin dryness. Although the precise chemical mechanisms underlying the anti-inflammatory effect of EEOs are not fully understood, some researchers attributed this activity to the presence of monoterpenes, especially 1,8 cineole, which is a powerful inhibitor of cytokine release (Moreira et al. 2022). Prior studies have illustrated that the main flavonoids in EEOs leaf offer several advantages for skin health (Aswandi et al. 2023).These significant findings strongly advocate for further investigation for anti-aging skincare.

### Wound healing effects

For thousands of years, traditional Aboriginal medicine has used ointments containing EEOs to help in wound healing (Chandorkar et al. 2021). Wound healing is a natural process that involves integrated and sequential processes to restore the function and integrity of damaged skin tissues. Several factors can impede this process, the most significant of which being microbial infection. Eucalyptus essential oil has wound-healing capabilities (Fayez et al. 2023). EEOs enhances wound healing through mechanisms such as angiogenesis, collagen deposition, granulation tissue development, epithelization, and wound contraction during the proliferative stage, according to Chandorkar et al. (2021). Furthermore, wound infection is a major cause of wound chronicity.

Previous studies indicated that bacteria may impede the process of wound healing at lower concentrations before infiltrating tissues, either by releasing toxins from living cells (exotoxins) or by causing cell death (endotoxins) (Robson 1997; Negut et al. 2018). Several studies have found that EEO has antimicrobial action against a variety of bacteria. Furthermore, (Bachir and Benali 2012) investigated EEO's antibacterial activity against two pathogens, *S. aureus* and *Escherichia coli*, the former being responsible for toxic shock syndrome, post-operative wound infection, and food poisoning, and the latter for urinary tract infections.

The chemical components responsible for antibacterial activity include 1,8-cineole, citronellol, citronellal, p-cymene, citronellyl acetate, eucamalol, linalool, α-terpinol, β-pinene, limonene, γ-terpinene, aromadendrene, and alloocimene. The essential oil contains 53.67% eucalyptol. It displayed considerable wound healing activity in the mice investigated, and the oil revealed a strong inhibition of inflammation (Nezhad et al. 2009; Tumen et al. 2017).

### Anti-acne effects

*E. globulus*, traditionally this plant claims the anti-inflammatory, analgesic and antimicrobial property. Moreover, EEOs is known to has effect on Propionibacterium acnes, the main causative agent for development of acne in human skin flora (Bhatt et al. 2011), as well as reducing inflammation and the formation of post acne scars. Inflammation is a sequence of repairing mechanisms characterized by increased blood flow to specific regions in response to detrimental stimuli such as irritation or pathogens. EEOs can decrease blood flow to the affected region and alleviate inflammation by enhancing blood circulation. Eucalyptol, when topically applied, possesses strong anti-inflammatory properties that aid in the rehydration and rejuvenation of the skin (Aswandi et al. 2023).

Moreover, the topical application of EEOs to treat acne is mainly due to geraniol, a compound with strong antibacterial properties that helps inhibit tyrosinase and reduce cytokines. Additionally, borneol, another compound in EEOs, acts as a natural antibiotic and anti-inflammatory agent. Other components like linalool, citronellol, and geraniol help restore skin elasticity and improve blood circulation, making EEOs effective for treating acne, skin aging, dry skin, and eczema. Moreover, several other mechanisms are involved in managing acne by EEO, including, it regulates sebum production, reduces the size of sebaceous glands to normal levels, and prevents secondary infections caused by other microbes. These actions address the primary causes of acne and help control its development. (Goodman 2006; Bhatt et al. 2011; Moreira et al. 2022). Also, its lipophilic properties may promote the skin’s microbiota, which is crucial for maintaining healthy skin (Wińska et al. 2019).

### Respiratory system health

Indigenous cultures across various regions have long recognized the therapeutic potential of *Eucalyptus* species. The leaves, in particular, have been employed for centuries to address respiratory ailments, including cough, cold, and congestion. Such ethnopharmacological knowledge provides a valuable foundation for scientific investigation into the pharmacological properties of EEOs (Aniwal et al. 2006).

A growing body of evidence supports the pharmacological properties of EEOs relevant to respiratory diseases. Various preclinical models have demonstrated their anti-inflammatory, antimicrobial, expectorant, and bronchodilator effects. These properties collectively contribute to the potential therapeutic benefits of EEOs in managing respiratory conditions.

### Upper respiratory tract infections

Upper respiratory tract infections (URTIs), including the common cold, sinusitis, and pharyngitis, are among the most prevalent human illnesses (Jain et al. 2001). Characterized by symptoms such as nasal congestion, cough, sore throat, and fever. URTIs significantly burden healthcare systems worldwide (Jain et al. 2001). Despite the availability of various over-the-counter and prescription medications, the search for effective and safe therapeutic options persists. EEOs has been traditionally utilized as a therapy for URTIs (Ben-Arye et al. 2011). Its potential benefits in alleviating symptoms and shortening the course of illness have prompted scientific investigation.

### Common cold and influenza

Common colds and influenza are extremely contagious viral diseases that mostly impact the upper respiratory system. The potential of EEOs to mitigate the severity and duration of cold and flu symptoms has been investigated. The observed effect can be linked to its antiviral and anti-inflammatory properties. It was previously reported that EEOs vapors possess a virucidal activity against the influenza A virus due to the inhibition of virus entry through reduced activity of its protein hemagglutinin, which is responsible for attachment to host cell receptors (Usachev et al. 2013; Vimalanathan and Hudson 2014). Recently, it was demonstrated in an invitro study that EEOs may show anti-coronavirus disease 2019 (COVID-19) by inhibiting both Angiotensin-converting enzyme II (ACE2) and lipoxygenase enzyme (LOX); the former acts as a functional receptor responsible for host cell entry of the SARS-CoV-2 virus, which causes COVID-19, while the latter was reported to have a pivotal role in the pathophysiology of COVID-19 through induction of pro-inflammatory leukotrienes and cytokines (Ayola-Serrano et al. 2021; Ak Sakallı et al. 2022). Moreover, molecular docking studies suggested that EEOs may interfere with viral replication by inhibiting Mpro enzyme (Muhammad et al. 2020; Panikar et al. 2021; El-Shiekh et al. 2024).

### Sinusitis

Sinusitis, an inflammation of the nasal sinuses, can lead to significant discomfort and impaired quality of life. EEOs possesses antimicrobial and anti-inflammatory properties, which suggest its potential as a therapeutic agent for sinusitis (Tesche et al. 2008; Djenane et al. 2011). Some clinical studies have shown promising results regarding symptom relief, improvement in mucus hypersecretion, and sinus drainage (Kehrl et al. 2004; Tesche et al. 2008). In another pharmacy-based, non-interventional survey, cineole, the main component in EEOs, resulted in substantial improvements in symptoms and quality of life of patients with favorable safety profiles (Werkhäuser et al. 2023).

### Pharyngitis

Pharyngitis, or sore throat, is a common symptom of various upper respiratory infections. EEOs’ anti-inflammatory effect could alleviate sore throat pain and discomfort via inhibition of COX-II (Juergens et al. 1998b; Lu et al. 2004; Ho et al. 2020). Moreover, EEOs can potentially eliminate possible pathogens, including bacterial and viral infections, as previously reported (Cermelli et al. 2008).

### Lower respiratory tract infections

Lower respiratory tract infections (LRTIs) encompass a range of conditions, including bronchitis and pneumonia. These diseases are characterized by inflammation and obstruction of the airways, leading to significant morbidity and mortality. Given the complex pathophysiology of LRTIs, there is an ongoing search for effective and safe therapeutic interventions. With its potential anti-inflammatory, antimicrobial, and bronchodilator properties, EEOs have attracted attention as a promising candidate for managing LRTIs.

### Bronchitis

Bronchitis is a respiratory condition characterized by inflammation of the bronchial tubes, the air passages connecting the trachea to the lungs. This inflammation leads to swelling and increased mucus production, resulting in symptoms such as coughing, wheezing, chest discomfort, and difficulty breathing (Kinkade and Long 2016). In a double-blinded and multi-centered study, cineole, the main ingredient of EEOs, showed anti-tussive properties and improved lung function in adult patients of acute bronchitis compared to the placebo group (Fischer and Dethlefsen 2013). Indeed, several studies have reported the possible mechanisms involved in the therapeutic effect of EEOs. In a previous study, eucalyptol (cineole) was found to inhibit mucus hypersecretion in COPD rats due to inhibition of overexpression of the MUC5AC gene, which encodes mucin protein, a key component in mucus (MUC5AC). Moreover, anti-inflammatory mechanisms were reported, including inhibition of toll-like receptor 4-induced transcription of pro-inflammatory gene via nuclear factor kappa B (NF-κB) dependent pathway as well as suppression of adhesion molecules expression such as intercellular adhesion molecule-1 (ICAM-1) thereby mitigating inflammatory cells infiltration (Zhao et al. 2014; Kennedy-Feitosa et al. 2016; Yu et al. 2018).

### Pneumonia

Pneumonia is a serious lung infection that bacteria, viruses, or fungi can cause. Cineole was previously reported to demonstrate anti-bacterial activity against various pathogenic strains causing pneumonia, such as *Staphylococcus aureus*, *Streptococcus pneumoniae*, *Klebsiella pneumoniae*, *Haemophilus influenzae*, as well as *Escherichia coli* and *Pseudomonas aeruginosa* (Salari et al. 2006; Bachir and Benali 2012; Warnke et al. 2013; Pereira et al. 2014; Ács et al. 2018; Miguel et al. 2018; Moo et al. 2021). In addition, EEOs alleviated influenza A-induced pneumonia in rats by inhibiting inflammatory responses and viral spreading in the lung and producing a synergistic effect with oseltamivir in such a model (Li et al. 2016). However, the role of EEOs in human respiratory syncytial virus as the most common cause of pneumonia in infants needs to be investigated.

### Obstructive lung diseases

EEOs, particularly its active component cineole (1,8-cineole), has shown promising therapeutic potential in the management of obstructive lung diseases such as asthma and chronic obstructive pulmonary disease (COPD). Cineole exhibits strong anti-inflammatory properties, which help reduce airway inflammation, a key feature of these conditions. Additionally, it aids in improving airflow and managing the symptoms.

### Asthma

Asthma is a chronic respiratory condition characterized by airway inflammation and hyperresponsiveness. In a previous double-blinded and placebo-controlled study, it was found that long-term treatment with cineole had a significant steroid-sparing effect in patients with steroid-dependent asthma by a mean reduction of 3 mg in the daily dose, suggesting that it may be a viable alternative for reducing steroid dose and use in asthma management due to its anti-inflammatory effects (Juergens et al. 2003). The beneficial outcome of using cineole in asthmatic patients was also reported in another study, as revealed by enhanced lung function and decreased dyspnea (Worth and Dethlefsen 2012). The mechanisms suggested for the anti-inflammatory effect included blocking the metabolism of arachidonic acid (Juergens et al. 1998a, 1998b), interacting with the transient receptor potential (TRPM8) channels (Caceres et al. 2017), mitigating mast cell degranulation (Nakamura et al. 2020), suppression of inflammatory cytokines production (Lee et al. 2016). Furthermore, a possible bronchodilation effect for the essential oil may explain its benefits in asthmatics besides the anti-inflammatory effect as revealed by the spasmolytic activity of EEOs and its main ingredient cineole on smooth muscles of bovine ilium and guinea-pig trachea, an effect that may be attributed to the calcium channel blocking activity (Coelho-de-Souza et al. 2005; Nozohour et al. 2022).

### COPD

Chronic obstructive pulmonary disease is a progressive respiratory disease characterized by persistent airflow limitation and chronic inflammation of the airways and lung tissue. It encompasses chronic bronchitis and emphysema, contributing to symptoms like chronic cough, sputum production, and breathlessness (Huertas and Palange 2011). Bronchodilators form the cornerstone of pharmacological treatment for individuals with COPD (Beeh 2016). The anti-inflammatory, bronchodilation, and mucolytic properties of EEOs suggest its potential to improve respiratory symptoms in COPD patients (Juergens et al. 2020). In a rat model of COPD, EEOs reduced the levels of TNF-α and IL-1ß in lung tissue as well as MDA levels, indicating an anti-inflammatory and anti-oxidant effects in alleviating the established emphysematous abnormalities (Wang et al. 2017). In a double-blinded, placebo-controlled study, cineole significantly reduced the severity, frequency, and duration of COPD exacerbations compared to placebo over a 6-month treatment period as well as showing an improvement in the symptoms of dyspnea except for dyspnea during exercise and also improving lung function (Worth et al. 2009).

### Gastrointestinal diseases

Eucalyptus oil has demonstrated potential therapeutic effects in gastrointestinal (GIT) diseases. The active component, cineole, exhibits significant anti-inflammatory and antimicrobial properties, which can help alleviate symptoms associated with GIT disorders. Eucalyptus oil is of interest for its potential to alleviate symptoms such as gastric inflammation, enhancing overall gastrointestinal effects. This part aims to explore existing evidence and shed light on the role of eucalyptus oil in gastrointestinal health.

### Peptic ulcer and gastritis

Gastritis refers to inflammation of the stomach lining. It can occur suddenly (acute gastritis) or develop gradually over time (chronic gastritis) (Rugge et al. 2020). Gastritis can cause erosions, or shallow sores, in the stomach lining, leading to deeper, larger open sores resulting in ulcer, the primary indication for peptic ulcer disease (Bereda 2022). The anti-inflammatory properties of eucalyptus oil, primarily due to its high eucalyptol content, play a crucial role in modulating the inflammatory responses associated with gastritis. Obviously, leukotrienes (LT) particularly LTC4 and LTD4 play a significant role in exacerbating gastric injury by ethanol and acids (Pihan et al. 1988). These lipoxygenase-derived metabolite of arachidonic acid contribute to the gastric injury and ulcer formation through promoting pepsin secretion and reducing transgastric potential difference (Pendleton et al. 1986). Moreover, LTC4 was reported to produce a vasoconstrictive effect in the gastric mucosa resulting in reduced mucosal blood perfusion (Yonei and Guth 1990). Based on the aforementioned data, inhibition of leukotrienes may explain at least in part the gastroprotective effect of eucalyptol in several studies regarding ulcer models (Santos and Rao 2001; Rocha Caldas et al. 2015; Tawfik et al. 2022). Moreover, its anti-inflammatory and anti-oxidant effects demonstrated a protective impact against trinitrobenzene sulfonic acid-induced colitis and aflatoxin B1-induced gastrointestinal injury in rats (Santos et al. 2004; Akinrinde et al. 2020). Another study demonstrated inhibitory action on *Helicobacter pylori,* the main causative agent for gastritis and peptic ulcer (Esmaeili et al. 2012).

### GI spasm

A previous study investigated the effect of eucalyptol on the spontaneous contractile activity of smooth muscles obtained from stomach of guinea pigs. The study findings revealed that eucalyptol exhibits either stimulatory or inhibitory actions depending on its concentration. Indeed, at low concentration (less than 2 × 10 – 5 M) eucalyptol exhibit stimulatory action, which authors attributed to activation of alpha-adrenoreceptors, on the other hand, at higher concentrations, eucalyptol stimulatory effect starts to decline until full suppression of spontaneous contractile activity occurs at a concentration of 4 × 10^–5^ M (Plamen et al. 2015). Moreover, the spasmolytic effect against spontaneous contractile activity of eucalyptol was comparable to papaverine (Plamen et al. 2015). Based on the previous data, eucalyptol holds the potential to act as anti-spasmodic in conditions when smooth muscle relaxation is required, however, due to limited data available, further studies are required.

**Fig. 5:**
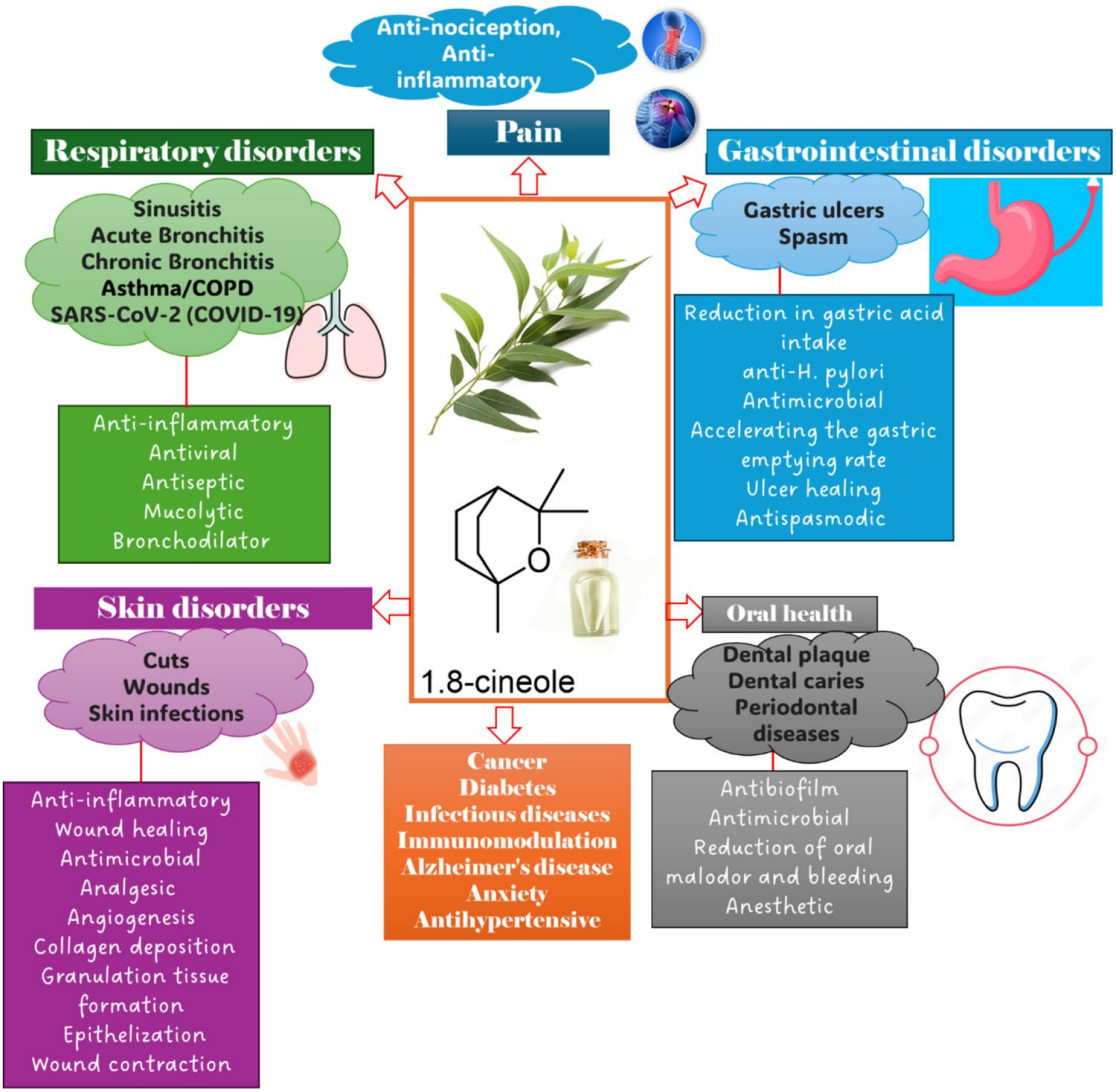
Therapeutic applications of the genus *Eucalyptus*

The therapeutic uses of *Eucalyptus* spp. for a range of illnesses and conditions will be explored in the section that follows, with an emphasis on the underlying molecular mechanisms of these applications. An overview of the several medicinal uses for *Eucalyptus* species can be found in Fig. 5.

## Conclusions

This review underscores the significant bioactive potential of EEOs, which possess a diverse range of antimicrobial, antiviral, and therapeutic properties. Key highlights include:

- **Diverse applications**: EEOs are cost-effective and environmentally friendly, finding uses in industries such as perfumery (perfume blending, fuel additives, soap making, and mining flotation agents), agriculture, and medicine.
- **Synergistic effects**: Mixtures of EEOs demonstrate enhanced efficacy compared to single oils, highlighting the importance of researching synergistic interactions.
- **Health benefits**: The primary component, 1,8-cineole, is linked to various health benefits, including antibacterial, anti-inflammatory, and anticancer effects, with potential applications in combating multi-drug resistance and serious diseases like COVID-19 and cancer.
- **Research gaps**: There is a pressing need for further investigation into the clinical efficacy, safety, and optimized formulations of EEOs, as well as the development of innovative drug delivery systems for respiratory diseases.

In summary, while EEOs present promising opportunities in healthcare and beyond, further research is essential to unlock their full potential and ensure safe, effective applications in clinical settings.

## Data Availability

No datasets were generated or analysed during the current study.
